# A unique presentation of hyponatremia and seizures in a 2-month-old child with cystic fibrosis: a case report

**DOI:** 10.1097/MS9.0000000000001048

**Published:** 2023-07-08

**Authors:** Sana Allouzi, Baraa Rihawi, Joud Allouzi, Mohammad I. Allouzi, Najwa Abdulrahman, Manar Abdullah

**Affiliations:** a Internal Medicine Department, Endocrinology Division; b Anesthesiology Department; c Dermatology Department, Aleppo University Hospital; d Faculty of Medicine; e Pediatrics Department, Faculty of Medicine, University of Aleppo, Aleppo, Syria

**Keywords:** case report, cystic fibrosis, hypochloremia, hyponatremia, seizure

## Abstract

Cystic fibrosis is an autosomal recessive disorder that causes a broad range of clinical findings and can present unusually as electrolyte disturbance, such as hyponatremia that causes seizures in children.

**Case presentation:**

We report a case of a 2-month-old infant who presented with an episode of grand mal seizure induced by hyponatremia and moderate episodes of milky vomiting after breastfeeding.

**Clinical discussion:**

Proper investigations showed normal cardiac and renal functions. Ultrasonography showed no pathological changes. Laboratory tests performed showed hyponatremia, mild hypokalemia, and hypochloremia. Urinary electrolyte results were normal. While broadening the scope of differential diagnosis in order to reach a final diagnosis, the sweat chloride level was elevated, which confirmed the diagnosis of cystic fibroses.

**Conclusion:**

We aim to share our case to keep cystic fibroses in mind as a differential diagnosis when dealing with hyponatremic seizures in children.

## Introduction

Highlights

- Cystic fibrosis (CF) is an autosomal recessive disorder; its most common presentations are respiratory and gastrointestinal; it can present unusually as electrolyte disturbance complications.
- CF should be considered in infants who have hypoelectrolytaemia and metabolic alkalosis.
- Pseudo-Bartter syndrome is associated with gastrointestinal disorders, but it may occur from extreme dermal losses of NaCl in CF.
- CF should be included in the differential diagnosis of children presenting with hyponatremia seizure.

Cystic fibrosis (CF) is an autosomal recessive disorder that causes a broad range of symptoms. The most common presentations are respiratory and gastrointestinal complaints. It can also present unusually as electrolyte disturbance such as hyponatremia^1^. However, hyponatremia is a widespread electrolyte disorder in pediatric patients. It is defined as a serum sodium level lower than 135 mEq/l, causing nonspecific mild symptoms to neurological manifestations^2^.

Hyponatremia is responsible for a vast number of afebrile seizures and causes more seizures in children in the first two decades compared with others^3^. The duration of hyponatremic seizures is longer in comparison to normonatremic seizures, with a higher incidence of status epileptics^4^. We report a case of CF that manifested with seizures induced by hyponatremia as an initial presentation, which makes the diagnosis a challenge in the absence of typical presentations.

This case report has been reported in line with the SCARE (Surgical CAse REport) Criteria^5^.

## Case presentation

A 2-month-old infant presented to the pediatric emergency department with complaints of recurrent moderate episodes of milky vomiting after breastfeeding with an episode of grand mal seizure that lasted for 2 min. His complaint of vomiting had begun 1 month earlier. The parents reported an episode of focal seizure and oculogyric crisis that lasted for 3 min, 3 days ago.

The parents did not report any episode of fever, rash, or other episodes of altered consciousness.

He was born at full term with an unremarkable prenatal, perinatal, and postnatal history. His parents are third-degree cousins, and his siblings are in good health. There is a family history of diabetes mellitus and no drug history. At the time of admission, the patient appeared mildly pale, and upon physical examination, his weight was 3.7 kg, the length was 53 cm, and the head circumference was 37 cm. His blood pressure was 90/50 mmHg, heart rate was 116 beats per minute, and temperature was 36.4°C. The auscultation of the chest and heart sounds was normal, and motor spiritual development was also normal and no stiff neck on examination. In addition, there was no visceral enlargement on palpation. On skin examination, there was no pigmentation, no rash and no signs of any injuries. The inspection of male sexual organs was also normal. Laboratory tests showed anemia (hemoglobin: 8.7 g/dl), hyponatremia (Na: 114 mEq/l) and mild hypokalemia (K: 3.4 mEq/l). White blood cells and C-reactive protein were normal. Kidney function tests and glucose were within normal limits. A biochemical urine test was normal. In addition, abdominal ultrasound and a chest X-ray showed no pathological changes. He required intravenous fluid therapy with potassium correction, and the sodium level was gradually corrected to avoid central pontine myelinolysis.

The follow-up after sodium correction was uneventful.

Further testing was needed for possible differential diagnoses. Laboratory tests showed hyperaldosteronism (53.8 ng/dl), hypochloremia (93.45 mmol/l) and normal levels of thyroid-stimulating hormone (1.05 mU/l), cortisol (12.03 μg/dl), parathyroid hormone (32.85 pg/ml), phosphate (44 mg/dl), magnesium (1.93 mg/dl), and calcium (10.5 mg/dl). Hypothyroidism and hypocortisolism were excluded. Urinary electrolyte results in random urine were also normal (Na: 35 mmol/l, Cl: 1.71 mmol/l, K: 16 mmol/l, Ca 10.14 mg/dl). Further discussion revealed that the infant had mucous diarrhea, and the suspected diagnosis of CF was confirmed with a sweat chloride level of 139 mmol/l. The infant was treated with standard therapy of CF and discharged from the hospital with a recommendation to avoid respiratory tract infections and hot exercises, and supporting fat-soluble vitamins.

## Discussion

CF is an autosomal recessive disease caused by a mutation in the cystic fibrosis transmembrane conductance regulator (CFTR) gene on chromosome 7. The most widespread mutation is the deletion of phenylalanine at codon 508. CFTR regulates chloride channels in epithelial cells. The defect causes reduced chloride ion secretion and increased sodium absorption, leading to high levels of sodium and chloride concentration in the sweat of patients with a diagnostic chloride level greater than 60 mmol/l in the sweat test^6^.

In a few cases in the literature, CF presented with recurrent vomiting, hypoelectrolytaemia and metabolic alkalosis, and primarily no chest complaints^7^.

In some cases, gastroesophageal reflux explains the recurrent vomiting in CF. Its findings were vomiting, recurrent pneumonia, and failure to thrive. In our case, the infant did not complain of respiratory symptoms^8^.

A correlation between hyponatremia, dehydration, and CF has been reported in many cases, accompanied with hypochloremia and metabolic alkalosis, especially in hot weather, with normal urine electrolyte concentration^9^.

However, hyponatremia is the most common electrolyte disturbance in infants, with significant complications such as hyponatremic encephalopathy, which can cause neurological harm. In healthy people, the body preserves normal serum sodium levels even with varying fluid intake by stimulating the kidney to excrete diluted urine and free water. The brain adapts to hyponatremia by intracellular electrolyte and organic osmolyte extrusion, which leads to neurological consequences. It is important to note that hyponatremic encephalopathy is more frequent in infants than in adults^10^. Children with CF are at higher risk of hyponatremia than healthy children, even in cool weather, due to the small amount of salt in breast milk and formula^11^.

However, hyponatremia in CF patients occurs because of excessive solute loss in comparison with fluid consumption, with low exchangeable sodium reservoir and dermal sodium losses accompanied by hypochloremia alkalosis^12^. Another mechanism of electrolyte disturbance is renal loss, such as Bartter and Gitelman syndrome. Bartter syndrome (BS) is a rare autosomal recessive disease that includes a defect in the thick ascending limb of the loop of Henle; its manifestations are polyhydramnios, dehydration, failure to thrive, and chronic kidney disease. The biochemical features of BS include hypercalciuria, nephrocalcinosis, and high concentration of urine chloride, which were not in our infant. On the other hand, Gitelman syndrome (GS) presents in late childhood or young adulthood and is asymptomatic or presents with muscle cramps, fatigue, and hypokalemic paralysis. The biochemical features of GS also include serum hypomagnesemia, hypokalemia, hypochloremia, hypocalciuria, and high concentration of urine chloride. However, the clinical features of BS and GS are different from CF, and renal loss of electrolytes in our case was excluded.

On the other hand, Pseudo-Bartter syndrome is due to extrarenal salt losses. It is associated with gastrointestinal diseases. It may occur from extreme dermal losses of NaCl in CF. The biochemical features include hyperaldosteronism, hypokalemia, metabolic alkalosis, hyponatremia, and low concentration of urine chloride^13^. The diagnostic clue of CF is the measurement of sweat chloride which defined the diagnosis in our case. Another possible cause of recurrent hyponatremia, growth failure, and high sweat chloride is dysfunction carbonic anhydrase 12 (CA12), which is a protein localized in the apical of the bronchiolar epithelia and basolateral membrane of the reabsorption duct of sweat glands. The loss of CA12 function causes an autosomal recessive disease characterized by chloride and sodium disturbance, and it should be kept in mind as a probable molecular cause of non-CF bronchiectasis^14^; however, the chest X-ray and the chest auscultation were normal in our patient.

In this manuscript, we report an infant with CF presenting with recurrent vomiting and seizures caused by hyponatremia without the common constitutional symptoms of CF. Our laboratory findings showed hypochloremia and hypokalemia in addition to hyponatremia. Renal losses of the electrolytes were excluded by urine electrolyte measuring electrolytes in the urine. Abdominal ultrasound ruled out hypertrophic pyloric stenosis as a possible cause. Endocrinology disorders were also excluded through tests. Few data on the frequency of seizures occurring in CF in infants have been reported in the medical literature, while some cases have been reported electrolyte decompensation in CF due to excess salts and fluid losses. Hyponatremia was presented in most of the new CF diagnoses; therefore, CF should be considered in the absence of possible causes of hypoelectrolytaemia, especially in infants^15^.

## Conclusion

In conclusion, CF could be presented in a variety of clinical manifestations, including electrolyte disturbance. Therefore, CF should always be considered as a possible diagnosis, particularly in countries where screening for CF is unavailable. Early diagnosis and treatment of CF can prevent or minimize the development of complications and improve the quality of life for affected individuals.

## Ethical approval

This study was not applicable for ethical approval.

## Consent for publication

Written informed consent was obtained from the patient’s parents for the publication of this case report. A copy of the written consent is available for review by the Editor-in-Chief of this journal on request.

## Sources of funding

The authors received no financial support for the research, authorship, and publication of this article.

## Author contribution

S.A. and M.A.: contributed to the conception and design of the work; B.R. and N.A.: contributed to data collection; J.A. and M.A.: contributed to the writing of the manuscript; M.A., S.A. and B.R.: contributed to the critical revision of the article. All authors read and approved the final manuscript.

## Conflicts of interest disclosure

The authors declare no potential conflicts of interest with respect to the research, authorship, and publication of this article.

## Research registration unique identifying number (UIN)

1. Name of the registry: not applicable.
2. Unique identifying number or registration ID: not applicable.
3. Hyperlink to your specific registration (must be publicly accessible and will be checked): not applicable.

## Guarantor

Sana Allouzi.

## Data availability statement

Data sharing are not applicable.

## Provenance and peer review

Not commissioned, externally peer-reviewed.

## Presentation

Not applicable.

## Acknowledgements

The authors thank Dr Abdulrahman Yousef Khaled and Dr Haya Almohammad for their support during this work.
